# p16/Ki-67 co-expression associates high risk human papillomavirus persistence and cervical histopathology: a 3-year cohort study in China

**DOI:** 10.18632/oncotarget.11705

**Published:** 2016-08-30

**Authors:** Lu-Lu Yu, Hui-Qin Guo, Xiao-Qin Lei, Yu Qin, Ze-Ni Wu, Le-Ni Kang, Xun Zhang, You-Lin Qiao, Wen Chen

**Affiliations:** ^1^ Department of Cancer Epidemiology, National Cancer Center, Cancer Hospital, Chinese Academy of Medical Sciences and Peking Union Medical College, Beijing, PR China; ^2^ Department of Pathology, National Cancer Center, Cancer Hospital, Chinese Academy of Medical Sciences and Peking Union Medical College, Beijing, PR China; ^3^ National Office for Maternal and Child Health Surveillance of China, West China Second University Hospital, Sichuan University, Chengdu, PR China

**Keywords:** HPV persistent infection, p16/Ki-67 co-expression, cervical cancer screening

## Abstract

**Purpose:**

To evaluate the association of p16/Ki-67 co-expression and persistence of high-risk human papillomavirus (HR-HPV) infection as well as cervical abnormalities.

**Methods:**

We performed a 3-year cohort study among which 2498 Chinese women aged 25 to 65 years were screened by different HPV tests in 2011. 690 women who were positive at any of the tests and a random sample of 164 women with all negative results received colposcopy, cervical specimens for cobas HPV test (Roche diagnostics) were collected before colposcopy; of this group, 737 cervical specimens were collected to perform cobas, Liquid-based cytology, HPV E6 test (Arbor Vita Corporation) and p16/Ki-67 dual staining (Roche diagnostics) in 2014. Colposcopy and biopsies was performed on women with any abnormal result.

**Results:**

Compared to women without HR-HPV persistent infection, women in the HR-HPV persistence group had a higher risk of p16/Ki-67 positive, with an adjusted Odds Ratio(OR) and 95% confidence interval (CI) of 6.29 (4.07-9.72); moreover, adjusted odds ratio for women who had HPV16/18 persistent infection was nearly 4-folder higher than women with other 12 HR-HPV persistent infection (adjusted OR = 17.15, 95% CI: 7.11-41.33 vs adjusted OR = 4.68, 95% CI: 2.89-7.58). Additionally, p16/Ki-67 positivity rate significantly increased with the severity of the cytological and histological abnormalities, and resulted strongly associated with a CIN2+ diagnosis (OR = 16.03, 95% CI: 4.46-57.59).

**Conclusions:**

p16/Ki-67 co-expressions associated strongly with HR-HPV persistence, especially with HPV16/18, and the presence of a CIN2+ lesion. Therefore, p16/Ki-67 could be considered as a suitable biomarker for cervical cancer screening, particularly in HPV-based screening programs.

## INTRODUCTION

The rates of cervical cancer have been greatly reduced by organized cytology screening programs in developed countries. In China, where efficient screening strategies are lacked, the burden of cervical cancer remains high with an estimation of 98,900 incident cases and 30,500 deaths in 2015[1]. The establishment etiologic association between cervical cancer and human papillomavirus (HPV) [2] drove the renovation of various cervical screening methods, especially high-risk HPV (HR-HPV) DNA testing, which has been evaluated as a replacement of Pap test in primary screening settings[3,4]. At the beginning of young women's sexual activity, HPV infection is extremely common, but more than 90% of the infections are transient and can be cleared in 1-2 years [5]; only a small proportion will progress and develop to invasive cervical cancer [6]. Since a single detection of HPV DNA cannot distinguish clinically relevant persistent infections from transient infections, the search of specific markers related to HPV persistence is urgently needed.

HR-HPV E6 and E7 oncoproteins are considered essential for the development of cervical cancer in persistent HPV lesions by interacting with p53 and pRB tumor suppressor proteins, which play an important role in the regulation of normal cell cycle [7]. p16^INK4a^ (p16) protein is a negative regulator of proliferation in normal cells though downregulating the activity of cyclin-dependent kinase(CDK) 4 and CDK6 once the pRB has been inactivated. HPV E7 can promote cell cycle progression by inactivating pRB, resulting in the overexpression of p16 in affected cells. Ki-67 is a nuclear protein and a cellular proliferation marker. The expression of p16 and Ki-67 is mutually exclusive in normal cells. The simultaneous detection of both proteins within a cell would be indicative of deregulation of the cell cycle caused by HPV oncoproteins expressed mostly during viral persistence, which suggests a possible relationship between p16/Ki-67 co-expression and HPV persistent infection. However, no study has directly proven such an association to date.

Here we used the CINtec PLUS Cytology test (Roche Tissue Diagnostics/Ventana Medical Systems, Inc., Tucson, AZ, USA) targeted for detection of p16/Ki-67 co-expression to investigate whether the presence of p16/Ki-67 associates with HR-HPV persistence as well as histology in a clinical study cohort in China.

## RESULTS

### Study participants description

**Figure 1:**
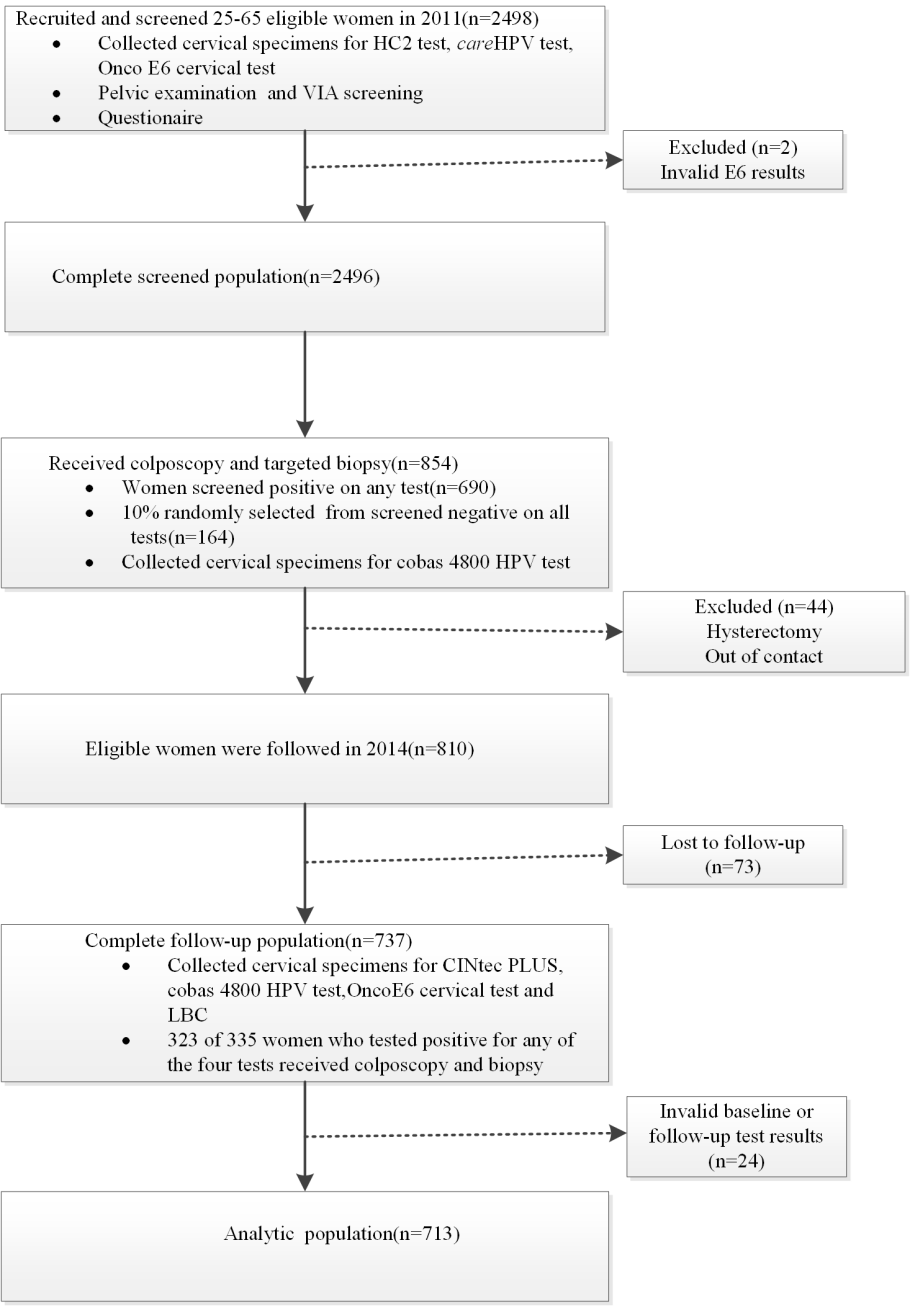
Flow diagram showing procedures involved in every step of the study

As shown in Figure 1, 2498 women were recruited in the study in 2011, of which 2496 (99.9%, 2496/2498) were eligible and had valid test results. 725 (29.0%, 725/2496) women tested positive by at least one of the screening tests and were referred to colposcopy; 1771 (71.0%, 1771/2496) women tested negative for all the tests, and 174 (9.8%, 174/1771) of those were also referred to colposcopy. 690(95.2%) of 725 and164 (94.3%) of 174 referral women completed the colposcopy and biopsy, and a total of 854 specimens were submitted to the cobas HPV test. In 2014, 810 of 854 referral women were to be followed. Of the 854 referral women, 23 (2.7%, 23/854) women with high grade cervical neoplasia were excluded due to cervical treatment after pathological diagnosis at baseline and 21(2.5%, 21/854) women were excluded for out of contact.737 (91.0%, 737/810) women completed the follow-up, 402 (54.5%, 402/737)women who tested negative for all four screening tests were considered negative for histological diagnosis ; 335(45.5%, 335/737) women tested positive for any of the four tests were referred to colposcopy and 323(96.4%) of 335 referral women completed the colposcopy and biopsy.713 (88.0%, 713/810) with valid baseline and follow-up HPV and p16/Ki-67 test results were included for analysis. 701(98.3%) of 713 women had valid histological diagnosis in the analysis group.

### HR-HPV infection at baseline and follow-up

The baseline and follow-up HR-HPV positive rates for women included in this study are shown in Table 1. 257(36.0%, 257/713) and 181(25.4%, 181/713) women were positive for HR-HPV at baseline and follow-up, 124(17.4%, 124/713) had HR-HPV persistent infection. The positive rates for HPV 16/18 were 12.9% (92/713) and 7.0% (50/713) at baseline and follow-up, and 33 (4.6%, 33/713) women had HPV16/18 persistent infection. 29.3 % (209/713) and 21.3% (152/713) women tested positive for other HR-HPV genotypes except HPV 16/18 at baseline and follow-up, 91(12.8%, 91/713) among them had persistent infection.

**Table 1:** Numbers and positive rates of HR-HPV infection among women included in this study

	HR-HPV positivity	
	N	%
Baseline		
HR-HPV	257	36
HR-HPV≠ HPV16/18	209	29.3
HPV 16/18	92	12.9
Follow-up		
HR-HPV	181	25.4
HR-HPV≠ HPV16/18	152	21.3
HPV 16/18	50	7
HPV persistence		
HR-HPV persistence	124	17.4
HR-HPV≠ HPV16/18 persistence	91	12.8
HPV 16/18 persistence	33	4.6

### Demographic and baseline characteristics of women grouped by p16/Ki-67 co-expression

Table 2 shows the baseline demographic characteristics for women in the p16/Ki-67 positive and negative groups. There were no differences between two groups for marital status, average annual income, second-hand smoking, drinking, and number of family member, lifetime sexual partners, age at first intercourse, age at menarche, contraception method, history of trichomonas infection / vaginomycosis/cervicitis, as well as family history of cervical cancer. p16/Ki-67 expression varied significantly by ages at baseline, educational level, parities and menopause.

**Table 2:** Baseline demographic characteristics of women included in this study by p16/Ki-67 expression status

Characteristics	p16/Ki-67 negative	p16/Ki-67 positive	*P*-Value
	*n* = 556	*n* = 157	
Age at baseline(Year)			
Continuous	45.9±7.8	50.8±8.0	<0.001
≤45	291(52.3%)	47(29.9%)	<0.001
≥46	265(47.7%)	110(70.1%)	
Marital status			
Married/ Cohabitation	541(97.3%)	149(94.9%)	0.133
Divorced / Widowed	15(2.7%)	8(5.1%)	
Education level			
Primary school or below	259(46.6%)	92(58.6%)	0.008
Middle school or above	297(53.4%)	65(41.4%)	
Average annual income(RMB)			
≤6000	350(64.1%)	105(68.2%)	0.349
>6000	196(35.9%)	49(31.8%)	
Second-hand smoking			
No	357(64.2%)	102(65.0%)	0.861
Yes	199(35.8%)	55(35.0%)	
Drinking			
No	527(94.8%)	149(94.9%)	0.952
Yes	29(5.2%)	8(5.1%)	
No. of family member			
≤4	303(54.5%)	90(57.3%)	0.529
≥5	253(45.5%)	67(42.7%)	
Lifetime sexual partaers			
1	509(91.5%)	146(93.0%)	0.558
≥2	47(8.5%)	11(7.0%)	
Age at first intercourse(Year)			
≤22	367(66.0%)	108(68.8%)	0.514
≥23	189(34.0%)	49(31.2%)	
Age at menarche			
≤16	377(67.9%)	99(63.5%)	0.295
≥17	178(32.1%)	57(36.5%)	
Parities			
≤2	358(64.4%)	69(43.9%)	<0.001
≥3	198(35.6%)	88(56.1%)	
Menopause			
No	392(70.5%)	74(47.1%)	<0.001
Yes	164(29.5%)	83(52.9%)	
Contraception method			
No	20(3.6%)	1(0.6%)	0.060
Yes	536(96.4%)	156(99.4%)	
History of trichomonas infection			
No	466(85.3%)	137(87.8%)	0.434
Yes	80(14.7%)	19(12.2%)	
History of vaginomycosis			
No	466(85.7%)	140(90.3%)	0.132
Yes	78(14.3%)	15(9.7%)	
History of cervicitis			
No	377(67.8%)	115(73.2%)	0.193
Yes	179(32.2%)	42(26.8%)	
Family history of cervical cancer			
No	534(96.0%)	151(96.2%)	0.939
Yes	22(4.0%)	6(3.8%)	
HR-HPV persistence			
HR-HPV none-persistent	502(90.3%)	87(55.4%)	<0.001
HR-HPV persistent	54(9.7%)	70(44.6%)	
HR-HPV persistence			
HR-HPV none-persistent	502(90.3%)	87(55.4%)	<0.001
HR-HPV persistent ≠ HPV16/18	47(8.5%)	44(28.0%)	
HPV 16/18 persistent	7(1.3%)	26(16.6%)	

### p16/Ki-67 co-expression and HR-HPV persistence

All the participants were divided into different groups according to HPV infection status (Table 3). Compared to HR-HPV non-persistent group, p16/Ki-67 co-expression in HR-HPV persistent group was significantly higher, with an odds ratio (OR) and 95% CI of 6.29(4.07-9.72) after adjustments for age at baseline, education level, parities, menopause; furthermore, adjusted odds ratio for women who had HPV16/18 persistent infection was 17.15 (95% CI: 7.11-41.33), which is nearly 4-folder higher than women with other 12 HR-HPV persistent infection (OR=4.68, 95% CI: 2.89-7.58).

**Table 3:** Crude and adjusted OR and 95% CI for the associations between HR-HPV persistence and p16/Ki-67 expression

HR-HPV persistence	subjects in analysis	Crude		Adjusted ^b^	
		OR	95%CI	OR	95%CI
HR-HPV none-persistent	589	-	-	-	-
HR-HPV persistent	124	7.48	4.91-11.40	6.29	4.07-9.72
HR-HPV persistent ≠ HPV16/18	91	5.40	3.38-8.64	4.68	2.89-7.58
HPV 16/18 persistent	33	21.43	9.02-50.90	17.15	7.11-41.33

Abbreviations: OR, odds ratio; CI, confidence interval. Crude and adjusted OR (95% CI): from unconditional logistic regression. Adjusted variables include age at baseline, educational levels, parities and menopause.

### p16/Ki-67 co-expression in different cytological/histological category

As shown in Table 4, the positivity rate for p16/Ki-67 in NILM, ASCUS, ASC-H, LSIL and HSIL was 16.8% (96/570), 25.3% (21/83), 70.0% (7/10), 62.5% (20/32) and 83.3% (5/6), respectively. There were 640 (91.3%, 640/701) women with negative histology, 46 (6.6%, 46/701) with CIN1, 11(1.6%, 11/701) with CIN2 and 4 (0.6%, 4/701) with CIN3, the corresponding p16/Ki-67 dual staining positivity was 17.3% (111/640), 56.5% (26/46), 72.7% (8/11) and 100.0% (4/4), respectively. Notably, all the HSIL and CIN3 cases tested positive for p16/Ki-67, p16/Ki-67 positivity increased significantly with cytology or histology severity in the whole population, and in women who were positive for HR-HPV (All *P_trend_* <0.001).

**Table 4:** Distribution of p16/Ki-67 positivity by cytological/histological report and HPV infection at follow-up

Category	p16/Ki-67 positivity *n* (%)		
	HR-HPV negative	HR-HPV positive ^b^	Total ^a^
All	64/528 (12.1)	85/173 (49.1)	149/701 (21.3)
Cytological diagnosis			
NILM	55/459(12.0)	41/111 (36.9)	96/570 (16.8)
ASCUS	4/55 (7.3)	17/28 (60.7)	21/83 (25.3)
ASC-H	1/3 (33.3)	6/7 (85.7)	7/10 (70.0)
LSIL	3/9 (33.3)	17/23 (73.9)	20/32 (62.5)
HSIL	1/2 (50.0)	4/4 (100.0)	5/6 (83.3)
Histological diagnosis			
normal	56/514 (10.9)	55/126(43.7)	111/640 (17.3)
CIN1	8/14 (57.1)	18/32 (56.3)	26/46 (56.5)
CIN2	0/0	8/11(72.7)	8/11 (72.7)
CIN3	0/0	4/4 (100.0)	4/4 (100.0)

Abbreviations: NILM, negative for intraepithelial lesion or malignancy; ASCUS, atypical squamous cells-undetermined significance; ASC-H (atypical squamous cells cannot exclude high-grade squamous intraepithelial lesion; LSIL, low-grade squamous intraepithelial lesion; HSIL, high-grade squamous intraepithelial lesion; CIN, cervical intraepithelial neoplasia; OR, odds ratio; CI, confidence interval.

a Chi-square for trend = 68.36, *p*-value < 0.001 (assessed for cytological category); Chi-square for trend = 69.87, *p*-value < 0.001 (assessed for histological category).

b Chi-square for trend = 20.16, *p*-value < 0.001 (assessed for cytological category); Chi-square for trend = 8.44, *p*-value = 0.004 (assessed for histological category).

### p16/Ki-67 co-expression in CIN2+ lesion

Table 5 shows the positivity for p16/Ki-67 related to the presence/absence of CIN2+ in the whole and HR-HPV positive participants with histological diagnosis. In the whole population, 12 cases of CIN2+ were p16/Ki-67 positive (80.0%, 12/15), while only 137 cases of CIN2- tested positive for p16/Ki-67 (20.0%, 137/686). p16/Ki-67 co-expression associated strongly with CIN2+ presence, the corresponding odds ratio and 95%CI was 16.03 (4.46-57.59). Moreover, 46.2% of CIN2- cases tested positive for p16/Ki-67 in the HR-HPV positive population, and the corresponding odds ratio and 95% CI between this biomarker and CIN2+ presence was 4.66 (1.27-17.15).

**Table 5:** Crude OR and 95% CI for the association between p16/Ki-67 positivity and CIN2+ presence in participants with a histological diagnosis

	CIN2-		CIN2+		OR (95%CI)
	*n*	%	*n*	%	
All (*n* = 701)					
p16/Ki-67 negative	549	80	3	20	-
p16/Ki-67 positive	137	20	12	80	16.03 (4.46-57.59)
HR-HPV positive group (*n* = 173)					
p16/Ki-67 negative	85	53.8	3	20	-
p16/Ki-67 positive	73	46.2	12	80	4.66 (1.27-17.15)

Abbreviations: CIN2+, cervical intraepithelial neoplasia grade 2 or higher.

## DISCUSSION

Recently, for women aged 25 years and older, the use of cobas HPV Test was approved by FDA for primary cervical cancer screening. This is of great importance to China, where cytology based screening has been proven difficult to implement in rural areas due to the lack of skilled cytopathologists. Notably, such cytology based or VIA based screening program which targets for screening 70% of 270 million Chinese women by 2015, resulted into the addition of 546,000 new HPV tests as announced by Chinese Government in 2014. However, as 80%–90% of women with a positive HPV test are transient infections and will not have concurrent disease, the challenge of using HPV testing for primary screening is to find complementary biomarkers that can discriminate clinically relevant persistent infection in screen-positive women. The simultaneous detection of p16 and Ki-67, which is commercially available now prompted our choice to evaluate its usage for such purpose.

We presented the first cohort study to examine the association between p16/Ki-67 co-expression and HR-HPV persistence. In a high risk screening population, we observed a significant higher positivity rate of p16/Ki-67 dual staining in HR-HPV persistence group. Compared to women without HR-HPV persistent infection, women in the persistent infection group had a higher risk of p16/Ki-67 positive, with an adjusted OR of 6.29 (95% CI: 4.07-9.72); moreover, adjusted odds ratio for women who had HPV16/18 persistent infection was 17.15 (95% CI: 7.11-41.33), which is nearly 4-folder higher than women with other 12 HR-HPV persistent infection (OR=4.68, 95% CI: 2.89-7.58). It may due to the higher carcinogenic potential of HPV16/18 oncoproteins than that of other carcinogenic HPV types [7,10]. Although a number of cross-sectional studies have investigated associations between p16/Ki-67 immunoreactivity and HR-HPV infections using cervical cytology or histology specimens, only one study reported the correlation between HR-HPV persistent infection and p16/Ki-67 expression in cervical biopsies [15]. However, this study found no predictive value of p16/Ki-67 at baseline on subsequent persistent infection with CIN1, which may be inconsistent with the present study, as differences between the two studies could be due to different study design, study population and methodology. Replications in future studies are needed to confirm these findings. In order to exclude the impactions of socioenvironmental factors including marital status, family circumstances, contraception method, parities, smoking, drinking and other factors like age, menopause, lifetime sexual partners, age at first intercourse/ menarche and history of other infections, which are likely to be involved in the process of HPV persistence and cervical carcinogenesis [16-19]and therefore may influence p16/Ki-67 expression, we compared them between two groups. Statistically-significant associations were found between age, parities, menopause, educational level and p16/Ki-67 co-expression, however, these factors were adjusted in multivariate models.

In the present study, to avoid the verification bias, four screening tests were used and women who tested positive for any of the tests were selected for colposcopy and directed biopsy, four-quadrant cervical biopsies and ECC were performed to maximize ascertainment of disease if no visible lesions were found under colposcopy. The histopathology of all biopsies was reviewed by an expert in CICAMS and all CIN cases were p16-supported. Since the establishment of a correlation between the biomarker expression and severity of neoplasia is the initial step to assess the potential use of a biomarker for cervical cancer screening[20], we evaluated p16/Ki-67 co-expression in different cytological and histological categories, and found that the test positivity of p16/Ki-67 increased significantly with disease severity. This is consistent with the previous studies though the number of CIN cases was limited and no cancer case was found in this study [12-14]. Importantly, all cases of CIN3 and 8 cases (72.9%, 8/11) of CIN2 displayed p16/Ki-67 co-expression, demonstrating the significant association between dual staining and CIN2+ presence (OR=16.03, 95% CI: 4.46-57.59). Notably, 3 cases of CIN2 with positive follow-up HR-HPV results tested negative for p16/Ki-67, perhaps due to the failure sampling since the 3 cases were also negative for LBC; another potential explanation is that not all CIN2 will progress, as 80% of CIN2 will regress to normal/CIN1 in 5 years without any intervention or treatment [21]. Moreover, 26 cases of CIN1 (56.5%, 26/46) were positive for p16/Ki-67. Interestingly, the majority of them tested positive for HR-HPV (69.2%, 18/26), which might be indicative of the possibility to progress. Follow-up of this cohort is needed to verify this hypothesis. Additionally, 49.1% (85/173) of women tested positive for dual staining in the follow-up HR-HPV positive group, with a high positive rate for CIN2+ cases (80%, 12/15). Based on the association between the biomarker and HR-HPV persistence evidenced in this study, p16/Ki-67 dual staining could be considered as an efficient triage method for HR-HPV positives. Furthermore, a series of studies has been conducted to evaluate the clinical performance of p16/Ki-67 dual staining in detection of cervical precancer and cancers [22-25]. One important requirement for the usage of a biomarker in the clinical practice is the consistence of positive interpretation standards. We used CINtec PLUS methodology in the current study, where whether a sample is positive is classified on the basis of simultaneous brownish cytoplasmic staining (p16) and red nuclear staining (Ki-67) in one or more cells, independent of cellular morphology. The recent published studies show that the interpretation of CINtec PLUS could be performed by staff not trained in the morphological interpretation of cytology after a short training phase [26,27]. It is of great significance to China, since the generalization of LBC is extremely difficult in low-resource rural areas.

Our analysis has several limitations. One consists in the definition of HPV persistence used in our study; there is wide variation in definition of HPV persistence in the literature[28-30], but repeat HR-HPV testing at 12 month is recommended in a meta-analysis[31], while the time interval between the two HPV tests in our study was three years; consequently, we cannot determine whether all cases of infection detected at follow-up was persistent infection or re-infection, and the conclusions may therefore have some bias. Another potential limitation is the CIN2+ cases are limited in numbers. This can be due to the small population size in the present study.

In summary, in a high-risk screening population, our study provides the first evaluation between presence of p16/Ki-67 and HR-HPV persistence. We observed p16/Ki-67 co-expression associated strongly with HR-HPV persistent infection, especially HPV 16/18. We found a strong association between dual staining and CIN2+ presence. Our results suggest that p16/Ki-67 is a suitable biomarker for cervical cancer screening, particularly in HPV-based screening programs. However, additional studies are needed to confirm these findings, particularly among populations with a large size.

## MATERIALS AND METHODS

### Study population

In 2011, a total of 2,498 women aged 25 to 65 years living in Xin-mi county were enrolled in a study named “Screening Technologies to Advance Rapid Testing for Cervical Cancer Prevention–Utility and Program Planning (START-UP)” Project based on the following criteria: 1) had a cervix; 2) had not been previously diagnosed with cervical cancer; 3) were not pregnant; 4) were physically able to undergo routine cervical cancer screening; 5) were able to provide informed consent. Women who were not married or never had sexual intercourse were excluded. Details on participants recruitment have been published elsewhere [32,33]. In 2014, part of these participants was followed. This study was registered with the U.S. National Institutes of Health and assigned the clinicaltrials.gov identifier: NCT01231945.The baseline study was approved by the institutional review boards (IRB) of the Cancer Institute/Hospital, Chinese Academy of Medical Sciences (CICAMS), of PATH (Program for Appropriate Technology in Health), and of the US National Cancer Institute; the follow-up study was approved by the IRB of CICAMS.

### Study procedures

Under START-UP, women were screened by 6 different screening tests in 2011: OncoE6^TM^ Cervical Test (Arbor Vita Corporation, Freemont, CA, USA) on a clinician-collected (cc) specimen, HC2 (QIAGEN) and careHPV testing (QIAGEN), both on a second cc and self-collected (sc) specimens, and visual inspection with acetic acid (VIA). One month later, women who tested positive for any of the 6 screening tests and an approximately 9.8% of randomly selected women who tested negative for all 6 tests (screen-negative women) were referred to colposcopy using a biopsy protocol as previously described[34]. Meanwhile, cervical specimens were recollected and tested for cobas HPV test (Roche Molecular Systems Inc., Pleasanton, CA). The referral population, except women who were histologically diagnosed as CIN2/3 and who received treatment at baseline as well as women who were out of contact, was followed and retested in 2014 by liquid based cytology (LBC), cobas HPV test (to assess status of HPV infection), OncoE6^TM^ Cervical Test, and p16 /Ki-67 dual staining (to assess status of p16 /Ki-67 co-expression). Women who tested positive for any of the four tests underwent colposcopy evaluation and biopsy using a protocol as mentioned above.

### HPV DNA testing

The cobas HPV test was performed according to the recommendations of the manufacturer. This assay, based on the amplification of target HPV DNA by PCR followed by nucleic acid hybridization, can detect 14 HR-HPV genotypes: HPV-16 and HPV-18 individually and the other 12 types pooled (31, 33, 35, 39, 45, 51, 52, 56, 58, 59, 66 and 68).

### p16 /Ki-67 dual staining

CINtec PLUS detects expression of p16 and Ki-67 as brown/cytoplasmic and red/nuclear reaction products, respectively. In accordance with the manufacturer's Instructions, sample is positive is classified on the basis of simultaneous brownish cytoplasmic staining (p16) and red nuclear staining (Ki-67) in one or more cells, independent of cellular morphology. Slides without any double-stained cells were called negative for p16/Ki-67 dual-stain cytology. All the slides were reviewed by a trained cytologist in CICAMS.

### Cytological and histological classification

Thin-layer cytology slides were prepared with ThinPrep Pap Test (Hologic Inc., Bedford, MA) and results were reported according to the Bethesda 2001 classification system. LBC results were considered negative when the result was negative for intraepithelial lesion or malignancy (NILM); all other results were considered positive and resulted in referral to colposcopy. All the cytological diagnoses were made by cytopathologists of CICAMS.

The histopathological diagnosis was provided by a senior CICAMS pathologist and the worst of the biopsies or surgical specimen was used for the final diagnosis in these analyses. Additional sections of all initial biopsy diagnoses with CIN were cut and tested for p16^INK4a^ by immunohistochemistry (Roche Tissue Diagnostics/Ventana Medical Systems, Inc., Tucson, AZ) as an additional adjudicator.

### Statistical analysis

Continuous and categorical variables were compared using t-tests and chi-square tests, respectively. The status of HR-HPV infection in the analysis was defined by the result of cobas HPV test. HR-HPV persistence was defined as HR-HPV positive both in 2011 and in 2014 via cobas HPV test. Associations between HR-HPV persistent infection and p16 /Ki-67 co-expression were examined using unconditional logistic regression models. Variables (age at baseline, education level, parities and menopause) associated p16 /Ki-67 co-expression were included as adjustment variables in logistic regression models. Chi square of trend for proportion was calculated to test linear associations between p16/Ki-67 dual staining and increasing severity of cytological and histological diagnoses. Odds ratios (OR) and 95% confidence interval (CI) by 2×2 tables were used to assess the association between p16/Ki-67 staining and histological outcomes. All P values less than 0.05 (two-sided) were considered to be statistically significant. SPSS 17.0 (SPSS Inc., Chicago, IL, USA) was used for the analyses.
